# Quantification of finger grasps during activities of daily life using convolutional neural networks: A pilot study

**DOI:** 10.1049/htl2.12080

**Published:** 2024-02-15

**Authors:** Manuela Paulina Trejo Ramírez, Callum John Thornton, Neil Darren Evans, Michael John Chappell

**Affiliations:** ^1^ School of Engineering University of Warwick Coventry United Kingdom of Great Britain and Northern Ireland; ^2^ Digital Human Research Team, Artificial Intelligence Research Center National Institute of Advanced Industrial Science and Technology Tokyo Japan

**Keywords:** biomechanics, neural nets, prosthetics

## Abstract

Quantifying finger kinematics can improve the authors’ understanding of finger function and facilitate the design of efficient prosthetic devices while also identifying movement disorders and assessing the impact of rehabilitation interventions. Here, the authors present a study that quantifies grasps depicted in taxonomies during selected Activities of Daily Living (ADL). A single participant held a series of standard objects using specific grasps which were used to train Convolutional Neural Networks (CNN) for each of the four fingers individually. The experiment also recorded hand manipulation of objects during ADL. Each set of ADL finger kinematic data was tested using the trained CNN, which identified and quantified the grasps required to accomplish each task. Certain grasps appeared more often depending on the finger studied, meaning that even though there are physiological interdependencies, fingers have a certain degree of autonomy in performing dexterity tasks. The identified and most frequent grasps agreed with the previously reported findings, but also highlighted that an individual might have specific dexterity needs which may vary with profession and age. The proposed method can be used to identify and quantify key grasps for finger/hand prostheses, to provide a more efficient solution that is practical in their day‐to‐day tasks.

## Graphical abstract

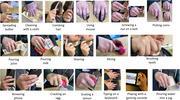

## 1 INTRODUCTION

Finger and fingertip injuries commonly occur due to work‐related incidents [1], particularly due to machinery and tool exposure during labour [2], and can result in digit or partial hand amputation. Approximately 68% to 78% of amputations in trauma cases occur in the upper limb, with finger amputations accounting for 90% of those cases [3]. Unfortunately, many individuals who undergo finger amputations are unable to continue in their original occupation, leading to a need for occupational change. Despite the frequency of finger amputations, advancements in finger prosthetics have been limited compared to full hand or lower‐limb prostheses [4]. To improve the design and functionality of prosthetic devices, it is essential to consider individual expectations and concerns [5]. Replicating human hand functionality as closely as possible is a key factor in achieving better prosthetic performance [6]. It is known that fingers work individually to achieve grasps. For example, the index finger and thumb work independently [7] and their movement varies from person to person. If we quantify the most frequent finger postures of a selected group of people, for example, people whose work involves the use of tools and machinery, then we can better understand individual finger synergies. The data obtained could inform on the kinematic parameters required for a prosthetic finger and the design would them help to restore function in a group that is highly exposed to injuries and amputations.

Previous studies have classified and created taxonomies of the variety of human hand grasps [8, 9, 10]. To the best of our knowledge, only a couple of studies have recorded and quantified such grasps during work tasks and activities of daily life (ADL): Battraw et al. [11] evaluated and categorized the hand activities of two children in their home environments and Zheng et al. [12] quantified grasp type and frequency in daily household and machine shop tasks. These studies were able to identify the most common grasps for their target subjects in specific environments and have shown that each target user has different preferences and manipulation strategies [13], which emphasizes the need for more studies evaluating grasps during ADL, including food preparation, eating, self‐maintenance, and even interaction with electronic devices.

Current studies on grasps during household, machining, and daily life tasks have certain limitations. They often rely on video recordings, which require manual identification of the grasps performed, making the process time‐consuming and prone to inaccuracies, as it is based on subjective judgement. Additionally, another limitation of these studies is that information on the full grasps executed is predominantly generated by observing the whole hand, due to the reliance of human judgement‐based analysis methods. This information is useful when needed for extrapolation to full hand function but can be limiting when trying to describe the specific role of a finger and its contributions to grasps and tasks. Our argument is that understanding individual finger motion is necessary for individual finger prosthetic design, whereas full hand function is useful mainly for full hand prostheses development. The main limiting factor is that when evaluating full hand kinematics, individual finger postures are evaluated with regards to the rest of the hand, which may not provide full information on how the finger works for finger prosthetic design.

Research indicates that the thumb, index, and little fingers demonstrate more individuated movements compared to the middle and ring fingers [7]. This shows the independence of finger motion reflecting hand neuromuscular architecture [14]. It is well known that multiple degrees of freedom of the hand are controlled by a lower number of actuators, meaning that there are intrinsic synergies that dictate hand and finger function [15].

In order to inform finger prosthetic design, this research aims to utilize recent developments in artificial intelligence for tracking and quantifying finger postures. While diverse options for finger prostheses exist in the market with varying levels of acceptability, adaptability, and workspace capabilities, no standards currently dictate how finger prosthetic design should meet these criteria and adapt to different needs [16]. By quantifying finger postures during ADL, it should be possible to design better prostheses that fulfil specific finger roles during specific tasks.

Therefore, the primary goal of our study was to evaluate if convolutional neural networks (CNNs) can be applied to recognize defined grasps during ADL for individual fingers. If true, this approach could offer a faster and more reliable analysis compared to video recordings, which often involve manual frame‐by‐frame labelling. Also, this approach takes advantage of the use of motion capture technology which is employed as it is the ‘gold standard’ for biomechanical evaluation of human movement.

The hypothesis of this research is that a subset of grasps can be used to describe the most crucial finger postures required during ADL. Hand postures can typically be characterized as a linear combination of a smaller number of synergies, and the same may apply to individual fingers. This implies that finger prosthesis design can be simplified through a dimensionality reduction approach, resulting in reduced size, weight, and cost.

The experiments performed used a motion capture system to record finger kinematic data during static grasps extracted from known taxonomies, which were transformed into custom RGB images fed into a CNN using transfer learning. Finger kinematics were also recorded during ADL. Trained CNNs identified grasps during such activities, allowing quantification of the most common grasps for each finger.

The presented research is intended as a proof‐of‐concept study. The objective of this letter is to explore and identify the most common finger postures related to grasps described in known taxonomies during ADL. We also hypothesized that the participant would exhibit similar sets of grasps to those reported in the literature, but with specific differences between fingers.

The results obtained from this method for identifying finger grasps during ADL will contribute to the design of larger cohort studies of users who could benefit from improved functional design of finger prosthetics based on real‐world ADL requirements data.

## 2 METHODS

The study received ethical approval from the University of Warwick Ethics Committee (ID: BSREC 138/21‐22). As the study will serve as a proof‐of‐concept evaluation of the potential of CNN to identify grasps, we recruited a single male, right‐handed participant (age = 34 years), who gave informed consent. The participant attended one data collection session.

### 2.1 Setup

Data collection took place at the University of Warwick Gait Laboratory. A motion capture system consisting of 12 MX‐T20 cameras (Vicon Motion Systems, Oxford, UK), collecting data at 125 Hz, was used.

**FIGURE 1:**
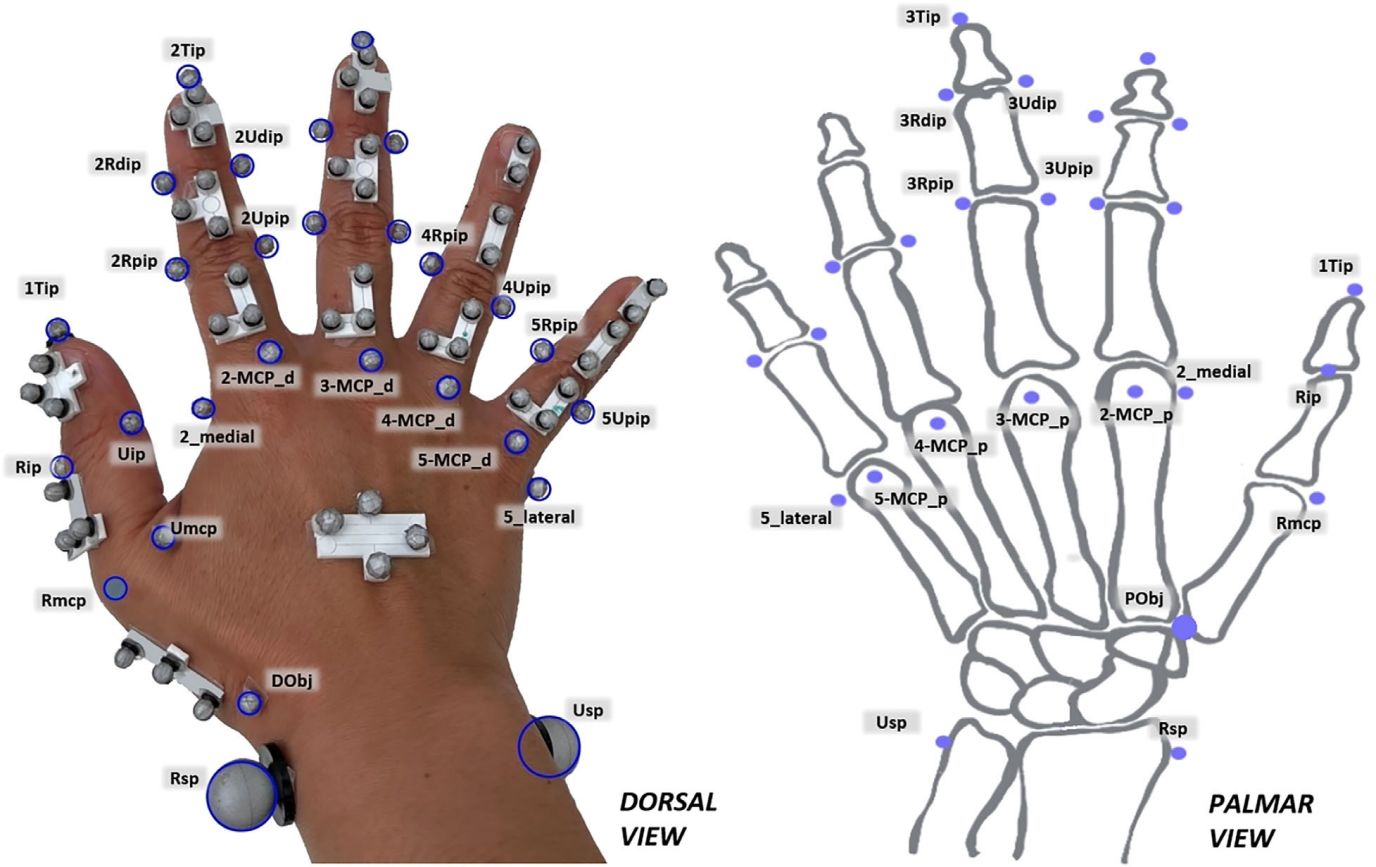
(a) Dorsal and (b) palmar view of the full marker set with calibration and tracking markers. Calibration markers are circled in blue.

A custom‐made marker set was used. The marker set was designed to evaluate tri‐dimensional finger biomechanical function using motion capture systems during ADL. A total of 88 markers were placed on the participant's right hand. This included 33 calibration markers, which were positioned on finger joint anatomical landmarks for the definition of rigid segments and removed after a static recording (see Figure 1 for the marker positions used). Non‐collinear markers were used to define the segments, with anatomical reference planes established using a right‐handed Cartesian coordinate system and a Cardan XYZ rotation sequence [17]. Distal interphalangeal (DIP) flexion, proximal interphalangeal (PIP) flexion, metacarpophalangeal (MCP) flexion and finger abduction/adduction angles were considered and recorded for each finger. Only the index, middle, ring, and little fingers were evaluated in this study.

A static trial was recorded with participant wearing all calibration and tracking markers, to capture the necessary kinematic segments. After removing the calibration markers, a zero‐degree baseline for the finger joints was established during the test by recording a calibration static trial using only tracking markers. Following the procedures described by Cook et al. [18] and Nataraj and Li [19], the participant utilized a flat, square block of wood, acting as a digit alignment device. In this position, the participant laid their hand flat with fully adducted fingers, while the thumb remained fully extended and adducted. Finger joint angles were recorded and averaged over 1 s for the normalization of all motion tasks and static grasps.

### 2.2 Training data

**FIGURE 2:**
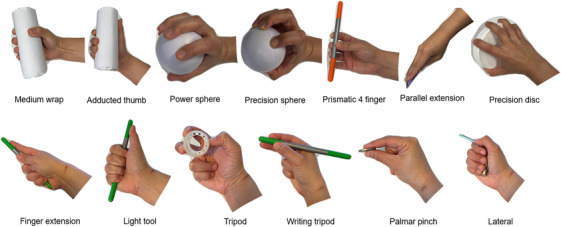
Selected grasps. The postures were used to train the CNN for each finger.

Thirteen grasps were extracted from the GRASP taxonomy [8] to be used for the training data set. The selection criteria included: (1) Grasps that allowed the recording of most finger segments with minimal overlapping or occlusion; (2) grasps where specific fingers were in contact with the object (relevant during Convolutional Neural Network training); and (3) commonly observed grasps during household and machining tasks [20]. The selected grasps are presented in Figure 2. The participant held the indicated object and maintained the desired grasp for 1 s without moving or changing position. The time was selected as suitable for the recording of intended grasp action of the hand when holding the indicated object. Ten occurrences per grasp were recorded.

### 2.3 Test data

**FIGURE 3:**
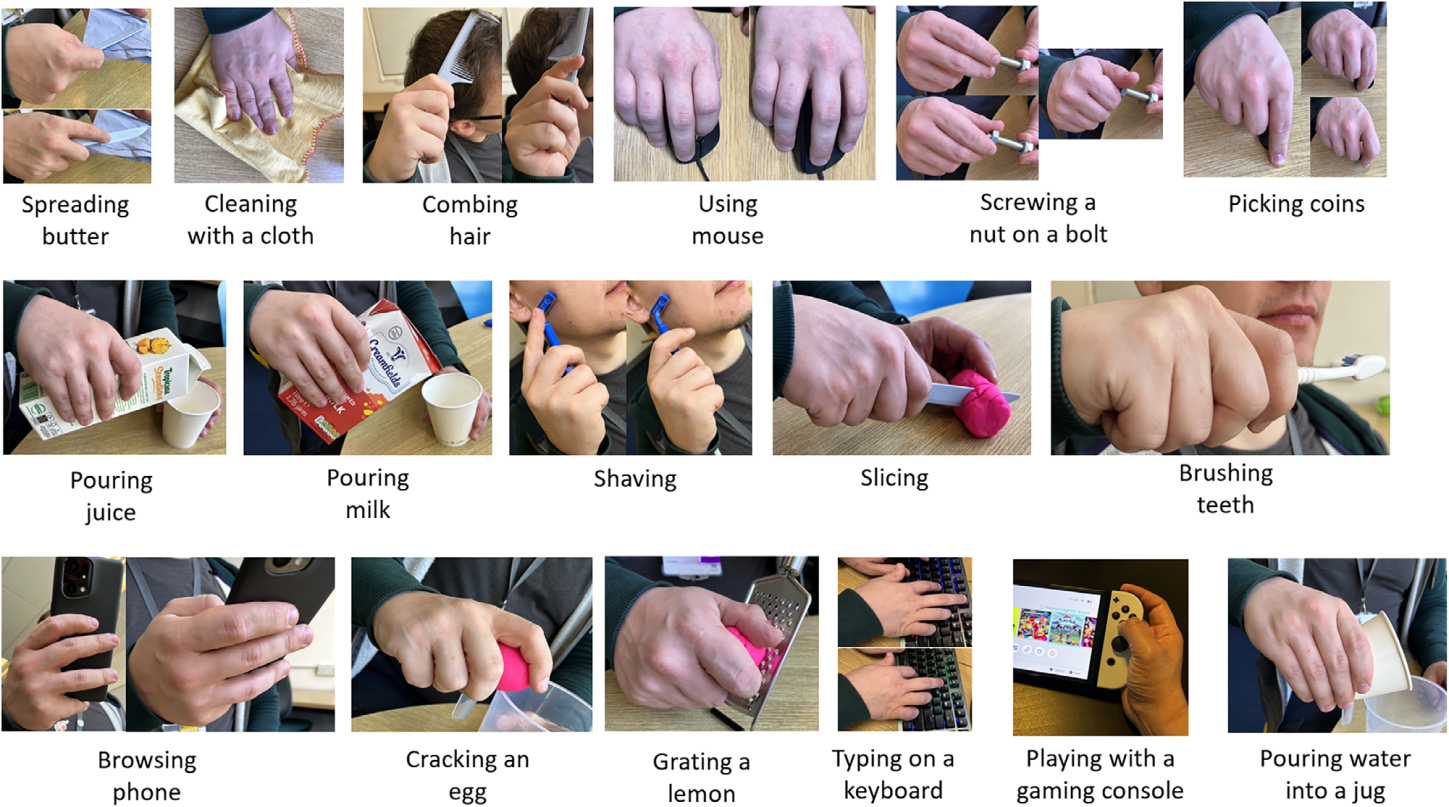
Images if the ADL tasks recorded. The participant used common household items to perform the tasks. The participant used for two of the tasks a Oppo X5 Pro smartphone and a Nintendo Switch OLED.

Following the recording of static grasps, the participant engaged in a series of ADL tasks, previously documented in the literature. These tasks encompassed eating and cooking tasks [21] along with related assessments such as Brief activity performance measure for upper limb amputees (BAM‐ULA) [22], the Sollerman Hand function test [23], and tasks aligned with the World Health Organization International Classification of Functioning, Disability and Health (ICF) [24]. These tasks were also representative of self‐maintenance, cleaning, and food preparation. Additionally, tasks associated with the use of technological devices were included, as these are increasingly becoming part of day‐to‐day lives. The devices chosen were a computer mouse, keyboard, mobile phone, and a videogame console (all chosen activities can be seen in Figure 3).

For each task, recordings were initiated from the moment the participant contacted the object, signalling readiness to perform the task and continued until the participant finished the task. The participant was instructed to perform the tasks in a natural manner. For tasks involving spreading butter, brushing teeth, and shaving, the participant was asked to simulate the tasks as closely as possible. In the case of tasks involving the use of a mouse and typing using a keyboard, the devices were connected to a tablet. The participant was then instructed to browse a webpage of his choice and compose an email, respectively. For tasks involving browsing on a mobile phone and playing with a videogame console, the participant was given 5 min to interact with Instagram and 9gag and play a selected videogame of their choice, in a typical manner.

### 2.4 Data processing

The raw marker trajectories were filtered in Vicon Nexus using a fourth‐order zero‐lag low‐pass Butterworth filter with a cut‐off frequency of 15 Hz. This filtering step aimed to eliminate displacement distortion that could lead to angle signal peaks during calculation. The resulting filtered marker trajectory data were then used to calculate finger joint angles using Vicon ProCalc. The angle data for all trials and tasks were further filtered using a zero‐lag fourth‐order low‐pass Butterworth filter with a cut‐off frequency of 5 Hz, following the recommendations outlined by Skogstad et al. [25], for hand motion tracking.

### 2.5 Creation of the training image dataset

Following the calculation of joint angles, the kinematic data were segmented per finger. The recorded data over 1 s were averaged for each posture for each finger and processed using bespoke MATLAB software.

**FIGURE 4:**
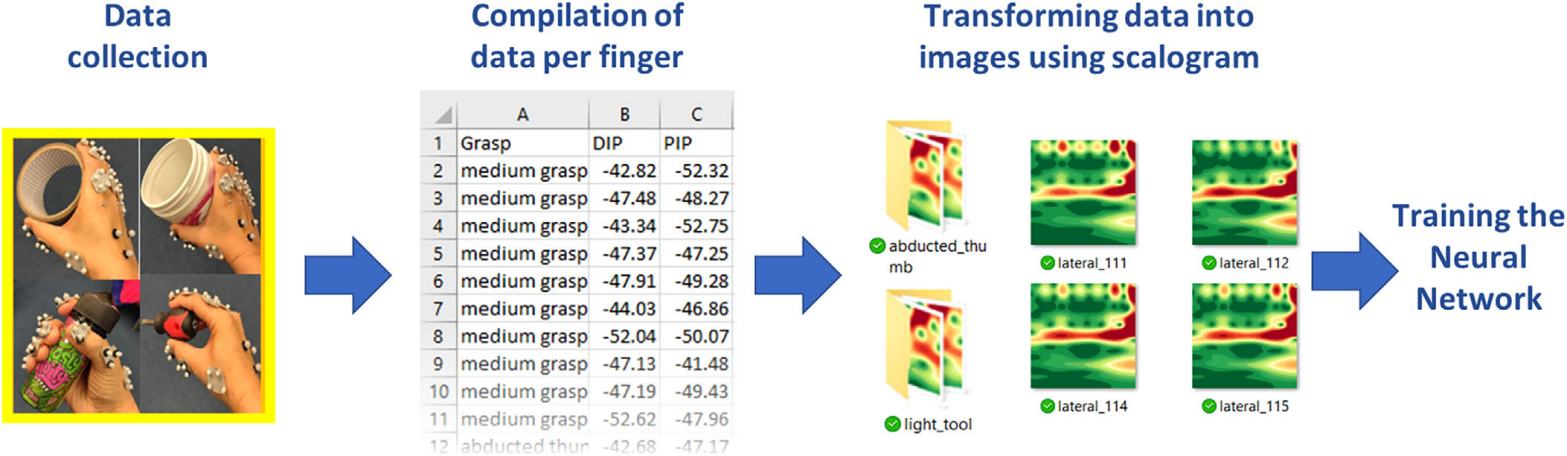
Creation of the training image data set. First, static grasps were recorded, then finger kinematics were calculated and transformed using a scalogram method. The images were used to train the GoogLeNet neural network.

To convert the finger joint angle data into images, the scalogram technique was employed. Initially, the kinematic data were transformed into a customized function. Subsequently, the data were transferred to the time‐frequency domain using a continuous wavelet transform to generate a scalogram image. The scalogram represents the kinematic data as an image composed of blobs, colours, loops, and lines. An example of how to build such images in Matlab is provided in [26]. The method was chosen due to its effectiveness in translating signals into images with discernible features for training and recognition tasks using CNNs [27]. To enhance the recognition of grasp differences, a custom colour map was utilized, emphasizing prominent image features. The process of converting finger kinematic data into images is illustrated in Figure 4. The resulting images were sized at 224 by 224‐pixels, aligning with the requirements of the selected CNN.

### 2.6 Transfer learning of the CNN

For the classification of grasp images, the GoogLeNet deep learning algorithm was used. GoogLeNet is a CNN network proposed by Christian Szegedy [28]. The network is 22 layers deep based on the Inception network architecture and pre‐trained using the ImageNet data set. The inception modules are built from multiple concatenated convolutional layers, which makes it very effective at extracting high‐level features for image recognition tasks. The network was chosen because it has shown better results in image classification compared to other networks (such as AlexNet) due to the number of layers utilized, displaying more efficient feature extraction for classification [29]. To incorporate a GoogLeNet network in our application transfer learning was used. Transfer learning is a machine learning approach that leverages the existing knowledge of a CNN to classify new images. This methodology requires a smaller amount of new data for training and benefits from the transfer of pre‐existing knowledge [30]. Using MATLAB's Deep Network Designer app, two layers at the end of the GoogLeNet network were replaced. These are the fully connected layer, combining primitive features into the specific patterns, and the output layer, which assigns a label to a test image [31].

Transfer learning was done by adapting the 22‐layer deep network GoogLeNet, subsequently trained for each finger using a subset of the grasps recorded. Using MATLAB's Deep Network Designer app, we modified the pre‐trained GoogLeNet network. The image input layer is set to take on 224 by 224‐pixel images in colour (RGB). Only the final fullyConnectedLayer and classificationLayer were replaced with the same layers but with different parameters, in this case the number of output classes (grasps) for each finger.

As not all fingers were in contact with the object or play a role during the selected grasps, a subset of grasps was selected for training each finger (see Table 1). A smaller subset of grasps was selected for the ring and little fingers, as these do not play a role in several grasps. For example, in writing tripod, these fingers flex completely to move away and allow the index and/or middle finger to grasp the object.

**TABLE 1:** Selected grasps for training the CNN for each of the fingers.

Grasps trained	Index	Middle	Ring	Little
Adducted thumb	✓	✓	✓	✓
Finger extension	✓	✓		
Lateral	✓	✓	✓	✓
Light tool	✓	✓		
Medium wrap	✓	✓	✓	✓
Palmar pinch	✓			
Parallel extension	✓	✓	✓	✓
Power sphere	✓	✓	✓	✓
Precision disc	✓	✓	✓	✓
Precision sphere	✓	✓	✓	✓
Prismatic 4 fingers	✓	✓	✓	✓
Tripod	✓	✓		
Writing tripod	✓	✓		

The training image data set used 10 images per grasp, using 30% of the data selected randomly for validation. Following the training parameters shown in [32], and follow‐up fine‐tuning, the networks were trained on a laptop equipped with an Intel i7‐11800 processor and a NVidia RTX3070 GPU graphics card. The training parameters for the network were set as follows: a base learning rate of 0.001, a mini‐batch size of 32, and training conducted for 100 epochs. The final training results for each network are summarized in Table 2.

**TABLE 2:** Final validation accuracy and loss of the networks for each finger.

Finger	Final validation accuracy	Final validation loss
Index	84.61%	0.40%
Middle	100.00%	0.04%
Ring	87.50%	0.32%
Little	100.00%	0.09%

### 2.7 Creation of the ADL image dataset

**FIGURE 5:**
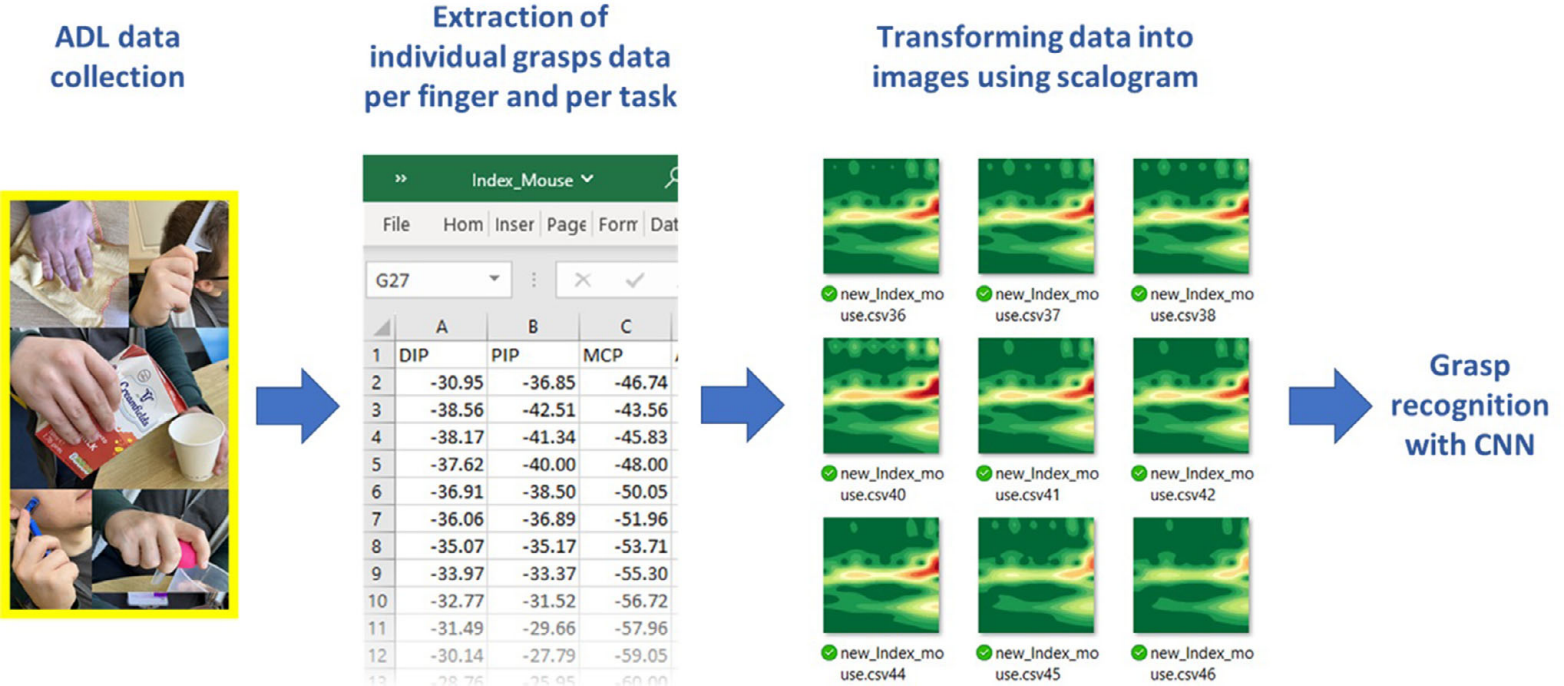
Example of the data extraction of one finger during ADL. Representative ADL were recorded and finger kinematics were used to create images via scalograms of each recorded static posture.

The ADL image dataset was created by extracting grasps for each activity. A ‘grasp’ during an ADL was defined as a consistent finger posture maintained across frames. To determine the boundaries of a grasp, the changes in joint angles between frames were examined. If there were no changes in joint angles greater than 1°, then this was considered a single grasp posture. The criteria chosen were deemed suitable as it allowed to reduce the number of grasps for testing during long ADL recordings but also was sensitive enough to average similar grasps where fingers were mostly static during tasks. On the other hand, if changes in angles exceeded one degree between frames, then this was considered a different grasp or posture. Frames where no joint angle changes over one degree occurred were averaged, representing a single grasp posture. The procedure for transforming finger kinematic data into images followed a similar process to the training dataset, as illustrated in Figure 5.

## 3 RESULTS

The neural network for each finger was used to identify trained grasps during ADL. The neural networks were able to recognize grasps used during the chosen activities. Total grasp occurrences and ADL occurrences per finger are shown in Tables 3, 4, 5, 6, 7, 8, 9.

**TABLE 3:** Index finger total grasp occurrence during ADL.

Grasp name	Number of instances	Percentage of data% instance
Adducted thumb	69	2.3
Finger extension	38	1.3
Lateral	42	1.4
Medium wrap	76	2.5
Palmar pinch	54	1.8
Power sphere	4	0.1
Precision disc	614	20.6
Precision sphere	1422	47.7
Prismatic 4 fingers	6	0.2
Tripod	545	18.3
Writing tripod	112	3.8

ADL, activities of daily living.

### 3.1 Index finger

The *precision sphere* grasp has the highest occurrence with 1422 instances, accounting for 47.7% of all grasps (see Table 3). Even though the *precision sphere* grasp dominated the percentage of data metric, this was due to the high number of instances identified when the participant used the mouse (see Table 4), but it was also present in other activities. This indicates that the index finger *precision sphere* grasp is commonly used during ADL and plays a significant role in manual dexterity. A similar case occurred with the *precision disc* grasp which was the second most frequent grasp appearing across different ADL. Other index finger grasps displaying lower occurrence rates, such as *prismatic 4 fingers*, *lateral*, and *power sphere*, are less commonly used and more specific to certain ADL which may require particular index finger positioning, such as picking up coins, screwing a nut on a bolt, or brushing teeth.

**TABLE 4:** Index finger grasp occurrence for each ADL.

ADL	Grasp name	Number of instances	Percentage of data% Instance
Spreading butter	Finger extension	1	12.5
	Precision disc	7	87.5
Cleaning with a cloth	Finger extension	16	69.6
	Precision disc	7	30.4
Combing hair	Precision sphere	17	65.4
	Tripod	9	34.6
Using a mouse	Adducted thumb	5	0.4
	Finger extension	2	0.2
	Medium wrap	16	1.4
	Precision disc	278	24.5
	Precision sphere	655	57.7
	Tripod	180	15.8
Screwing a nut on a bolt	Lateral	21	23.1
	Palmar pinch	9	9.9
	Tripod	18	19.8
	Writing tripod	43	47.3
Picking up coins	Adducted thumb	5	19.2
	Precision disc	1	3.8
	Prismatic 4 fingers	1	3.8
	Tripod	13	50.0
	Writing tripod	6	23.1
Pouring juice	Medium wrap	1	6.3
	Precision sphere	12	75.0
	Tripod	3	18.8
Pouring milk	Precision disc	8	27.6
	Tripod	21	72.4
Shaving	Adducted thumb	18	58.1
	Tripod	13	41.9
Slicing	Adducted thumb	2	8.7
	Precision disc	6	26.1
	Precision sphere	13	56.5
	Tripod	2	8.7
Brushing teeth	Adducted thumb	39	2.5
	Finger extension	19	1.2
	Lateral	21	1.3
	Medium wrap	59	3.8
	Palmar pinch	44	2.8
	Power sphere	4	0.3
	Precision disc	307	19.6
	Precision sphere	722	46.1
	Prismatic 4 fingers	5	0.3
	Tripod	284	18.1
	Writing tripod	63	4.0
Browsing on a phone	Precision sphere	1	100.0
Cracking an egg	Precision sphere	1	100.0
Grating a lemon	Palmar pinch	1	100.0
Typing on a keyboard	Tripod	1	100.0
Pouring water into a jug	Precision sphere	1	100.0
Playing with a console	Tripod	1	100.0

ADL, activities of daily living.

### 3.2 Middle finger

Among the identified grasps for the middle finger, the most frequently observed was the *light tool* grasp, which accounted for 29.5% of all instances (see Table 5), but this was mainly observed during one specific ADL (combing hair, see Table 6), whereas the second most identified grasp (*writing tripod*) was identified in four of the considered ADL. The results showed that the middle finger might provide a supportive role in grasps complimentary to index finger and thumb grasps, where more support is required, and where the index finger cannot provide enough stability and dexterity to adapt to object size or manipulation requirements.

**TABLE 5:** Middle finger total grasp occurrence during ADL.

Grasp name	Number of instances	Percentage of data% instance
Precision sphere	1	0.3
Adducted thumb	34	9.2
Finger extension	32	8.6
Lateral	15	4.1
Light tool	109	29.5
Medium wrap	13	3.5
Parallel extension	10	2.7
Precision disc	2	0.5
Precision sphere	20	5.4
Prismatic 4 fingers	36	9.7
Tripod	20	5.4
Writing tripod	78	21.1

ADL, activities of daily living.

**TABLE 6:** Middle finger grasp occurrence for each ADL.

ADL	Grasp name	Number of instances	Percentage of data% instance
Cleaning with a cloth	Parallel extension	4	
Combing hair	Adducted thumb	5	2.6
	Finger extension	5	2.6
	Lateral	7	3.7
	Light tool	109	57.4
	Medium wrap	4	2.1
	Parallel extension	1	0.5
	Prismatic 4 fingers	2	1.1
	Tripod	1	0.5
	Writing tripod	56	29.5
Using a mouse	Precision sphere	1	100.0
Screwing a nut on a bolt	Adducted thumb	7	15.9
	Medium wrap	8	18.2
	Precision sphere	12	27.3
	Tripod	6	13.6
	Writing tripod	11	25.0
Pouring juice	Tripod	1	100.0
Pouring milk	Precision disc	1	100.0
Shaving	Medium wrap	1	100.0
Brushing teeth	Adducted thumb	22	18.5
	Finger extension	27	22.7
	Lateral	8	6.7
	Precision sphere	7	5.9
	Prismatic 4 fingers	33	27.7
	Tripod	12	10.1
	Writing tripod	10	8.4
Browsing on a phone	Precision disc	1	100.0
Cracking an egg	Prismatic 4 fingers	1	100.0
Typing on a keyboard	Parallel extension	5	100.0
Pouring water into a jug	Precision sphere	1	100.0
Playing with a console	Writing tripod	1	100.0

ADL, activities of daily living.

It is worth noting that the data include a small number of instances for certain grasps, such as *precision sphere* (one instance) and *precision disc* (two instances). Therefore, while these grasps had a low overall occurrence, it is important to note their role during ADL for this particular participant—it might not be a significantly necessary component of middle finger function to achieve stable grasps. The remainder of the identified middle finger grasps were more evenly distributed among tasks.

### 3.3 Ring finger

Among the recorded grasps, the most prevalent ring finger grasp was the *prismatic 4 fingers*, which accounted for a significant proportion of instances, 42.9% (see Table 7). This was dominated by the number of instances identified for this grasp during the screwing a nut on a bolt ADL (see Table 8). The second most prevalent grasp (*lateral*) appeared in two ADL (shaving and brushing teeth). It seems that, in the case of the ring finger, certain postures tended to dominate finger dexterity during specific ADL. This may vary from task to task, as there were no grasps that commonly appeared during all of the ADL considered for the ring finger, indicating their occasional utilization during ADL.

**TABLE 7:** Ring finger total grasp occurrence during ADL.

Grasp name	Instance	% Instance
Adducted thumb	18	11.2
Lateral	62	38.5
Medium wrap	5	3.1
Power sphere	1	0.6
Precision disc	1	0.6
Precision sphere	5	3.1
Prismatic 4 fingers	69	42.9

ADL, activities of daily living.

**TABLE 8:** Ring finger grasp occurrence for each ADL.

ADL	Grasp name	Number of instances	Percentage of data% instance
Cleaning with a cloth	Power sphere	1	100.0
Using a mouse	Precision sphere	1	100.0
Screwing a nut on a bolt	Adducted thumb	2	7.7
	Lateral	9	34.6
	Medium wrap	2	7.7
	Prismatic 4 fingers	13	50.0
Pouring juice	Precision sphere	1	100.0
Pouring milk	Precision sphere	1	100.0
Shaving	Adducted thumb	16	55.2
	Lateral	11	37.9
	Medium wrap	2	6.9
Brushing teeth	Lateral	42	42.9
	Prismatic 4 fingers	56	57.1
Browsing on a phone	Precision sphere	1	100.0
Typing on a keyboard	Precision disc	1	100.0
Pouring water into a jug	Precision sphere	1	100.0
Playing with a console	Medium wrap	1	100.0

ADL, activities of daily living.

### 3.4 Little finger

The most frequently observed little finger grasp was the *power sphere* which accounted for a significant portion of instances at 36.7% (see Table 9). The second most prevalent grasp was the *lateral*, constituting 16.1% of instances. Both grasps only appeared in two of the ADL, whereas the other grasps were distributed over the remaining ADL, with the little finger *prismatic 4 fingers* grasp, appearing in only two instances during the shaving task (see Table 10).

**TABLE 9:** Little finger total grasp occurrence during ADL.

Grasp name	Number of instances	Percentage of data% instance
Adducted thumb	28	14.1
Lateral	32	16.1
Medium wrap	27	13.6
Parallel extension	22	11.1
Power sphere	73	36.7
Precision disc	15	7.5
Prismatic 4 fingers	2	1.0

ADL, activities of daily living.

**TABLE 10:** Little finger grasp occurrence for each ADL.

ADL	Grasp name	Number of instances	Percentage of data% Instance
Using a mouse	Parallel extension	11	91.7
	Precision disc	1	8.3
Screwing a nut on a bolt	Adducted thumb	4	28.6
	Lateral	1	7.1
	Power sphere	9	64.3
Pouring juice	Parallel extension	1	100.0
Pouring milk	Parallel extension	10	100.0
Shaving	Adducted thumb	15	17.6
	Medium wrap	10	11.8
	Power sphere	58	68.2
	Prismatic 4 fingers	2	2.4
Brushing teeth	Adducted thumb	9	11.8
	Lateral	31	40.8
	Medium wrap	16	21.1
	Power sphere	6	7.9
	Precision disc	14	18.4
Browsing on a phone	Medium wrap	1	100.0
Typing on a keyboard	Precision disc	1	100.0
Pouring water into a jug	Adducted thumb	4	5.8
	Medium wrap	19	27.5
	Parallel extension	14	20.3
	Precision disc	13	18.8
	Precision sphere	9	13.0
	Prismatic 4 fingers	10	14.5
Playing with a console	Adducted thumb	1	100.0

ADL, activities of daily living.

It is thought that the function of the little finger during tasks employing the *power sphere*, such as pouring juice, shaving and screwing a nut on a bolt, is to provide support for the other fingers. This can be argued practically during tasks involving large heavy objects or during meticulous tasks with smaller objects. Other grasps had very similar frequencies in total. Brushing teeth, combing hair, screwing a nut on a bolt and shaving were tasks where the most frequently occurring grasps were identified. The data show that some tasks require varied ring finger grasps to allow for small adjustments. This can be seen in transitions between finger postures from one grasp to the other, allowing the hand to adapt to task requirements and in‐hand manipulation of a small object/tool, for example, rotating the comb, toothbrush, and the razor during meticulous tasks.

## 4 DISCUSSION

This letter presents the first individual quantification of grasps for selected fingers during ADL. This quantification of finger grasps can help to prioritize finger postures for prosthesis design and further contribute to our understanding of hand function and grasp preferences during daily activities. The employed CNN were able to identify the most prevalent grasps for each finger studied.

The grasps with highest total percentage of frequency during this study were the *precision sphere* for the index finger (47.7%) and the *prismatic 4 fingers* for the ring finger (42.9%). The *medium wrap* and *precision disc* grasps were identified for all fingers. The *finger extension* grasp was found only on the index finger data, whereas the *lateral* grasp was observed for all fingers except the index finger, which shows the capabilities of the CNN to identify grasps present in specific fingers, which provides vital information on each finger movement strategy during ADL.

Most previous studies regarding hand function and grasps have focused on creating taxonomies and classifications of human hand function. To the best of our knowledge, there are only two previous reports quantifying grasps during machinist and house cleaning tasks. The first one is an investigation of the grasp types and frequencies of a professional housekeeper and a machinist [20]. The findings showed that the most common grasps differed between professions, which could indicate that grasping requirements vary between subject needs. There were similarities between our findings and the aforementioned studies of both the housekeeper and the machinist, as their most common grasps were present in our study for all fingers but in varying frequency. The most common grasps for the machinist, and in this study, were the *lateral pinch, light tool, tripod, medium wrap, finger extension*, and *thumb‐index* (palmar pinch) grasps. For the housekeeper, identified grasps in common were the *medium wrap, finger extension, power sphere, lateral, precision disc*, and *palmar pinch* grasps [33].The common grasps identified in our pilot study and the grasps from the housekeeper and the machinist might help to confirm the role of the hand and fingers during job‐specific tasks, which could also contribute to the design of job‐specific finger prostheses.

In this pilot study, identified grasps for the index finger, similar to those for the housekeeper, were the *finger extension, lateral, medium wrap, palmar pinch*, and *precision sphere* grasps, whereas *prismatic 4 fingers* and *writing tripod* were also observed for the index finger as for the machinist.

Common grasps observed for the machinist were mainly present in specific fingers. For example, the *light tool* grasp was only observed for the middle finger, accounting for approximately 30% of the total grasps identified. The *tripod* grasp accounted for a larger total frequency percentage for the index finger than for the middle finger (18.3% vs. 5.4%). These results confirm finger independence in the execution of job‐related tasks and ADL.

The second study quantifying grasps during ADL focused on children in their home environments. Study findings showed that rehabilitation and prostheses could benefit from age‐appropriate and activity‐specific task considerations to obtain better outcomes and increased functional independence [11]. The housemaid and machinist study suggested that six to nine grasps can account for approximately 80% of activities, whereas they found that seven grasps accounted for 90% or more of daily activities for children. In this study, two to six grasps accounted for more than 80% of activities for each finger. It is likely that by focusing on individual fingers we can better reduce kinematic complexity and focus on functional design for a specific finger prosthetic. For example, in comparison, six grasps accounted for 83% of total grasp occurrence for the middle finger whereas only two grasps accounted for 81% of the grasps for the ring finger. Specific fingers imply specific motor requirements to achieve a stable grasp during ADL. Their findings identified the *tripod, lateral pinch, parallel extension*, and *medium wrap* as the most frequent grasps required. In part, this agrees with our findings but also confirms the specificity of grasp requirements for different users and groups.

A quantitative taxonomy of human grasps based on electromyography and kinematic data was able to divide hand movements into five categories based on hand shape, finger positioning, and muscle activity [34]. Our identified common grasps per finger fall within the following identified categories: distal, spherical, and flat grasps. Such grasps generally focus on allowing the fingers to adapt to object size and shape, permitting precision tasks to be performed.

The main limitation of this current study lies in the small training and validation data sets and the lack of comparison with other identification methodologies using video recordings. On the other hand, this study's strengths lie in the innovative evaluation and fast, automated, recognition of grasps from ADL. Another limitation of our study is the limited amount of data collected as the experiment was designed to be a pilot, proof‐of‐concept study to evaluate if CNN are appropriate for the identification and quantification of grasps seen during ADL. Furthermore, additional training data need to be collected to evaluate CNN accuracy during classification. On the other hand, the collected data suggests that specific grasp strategies are required for different fingers. Consequently, more in‐depth group‐focused evaluations could highlight important grasp occurrences, which could improve the design considerations of rehabilitation interventions and assistive and prosthetic devices.

The methodology presented can be helpful in creating comprehensive special training data sets that can be used at a later stage to improve functional rehabilitation interventions. For example, the MusicGlove is a low‐cost device developed as a medium to guide hand exercise and quantifies movement recovery [35]. Such devices and therapies could be improved by quantifying finger postures and grasps stereotypical of a user group (in this case, musicians) to create targeted rehabilitation sessions that relate to patient needs and increase patient engagement during rehabilitation. As previous studies have shown, grasping strategies and the needs of users vary greatly with age and occupation.

With the initial results obtained from a single participant, we propose this methodology to facilitate finger kinematics and grasp recognition within ADL for larger cohorts and specific groups of users. Future applications include use in hospitals and improving current manual evaluations of video recordings. The method has shown potential for the recognition of grasps using machine learning, which could lead to further research in rehabilitation and prosthesis design. By quantifying the most frequently used hand/finger grasps, prosthetic design could be oriented to cover a specific range of needs when designing for a large group or to specialize functionality based on certain target users. Future studies would help to quantify stereotypical grasps in different prosthetic target groups; for example, manual workers, who are commonly exposed to hazards and who are highly affected following finger amputation. A prosthetic finger is required to perform the tasks observed in day‐to‐day life if it wishes to restore functionality as much as possible. Future evaluations and quantification of grasps for prosthesis design should go beyond household and self‐maintenance tasks, with consideration of the specific needs of the job/profession, age of the user [11], hobbies [33], and the use of modern technological devices [36].

## AUTHOR CONTRIBUTIONS

**Manuela Trejo Ramírez**: Conceptualization; investigation; methodology; validation; writing‐original draft. **Callum Thornton**: Conceptualization; investigation; methodology; writing‐original draft; writing‐review & editing. **Evans Neil**: Conceptualization; methodology; project administration; supervision; validation; writing‐review & editing. **Michaell Chappell**: Conceptualization; funding acquisition; methodology; project administration; resources; supervision; validation; writing‐review & editing.

## CONFLICT OF INTEREST STATEMENT

The authors declare no conflict of interest.

## Data Availability

The data set used for this study will be made available to researchers upon reasonable request.
